# Facile Synthesis and Characterization of Ag_3_PO_4_ Microparticles for Degradation of Organic Dyestuffs under White-Light Light-Emitting-Diode Irradiation

**DOI:** 10.3390/ma11050708

**Published:** 2018-04-30

**Authors:** Chi-Shun Tseng, Tsunghsueh Wu, Yang-Wei Lin

**Affiliations:** 1 Department of Chemistry, National Changhua University of Education, Changhua City, 500 Taiwan; sars124578@yahoo.com.tw; 2 Department of Chemistry, University of Wisconsin-Platteville, 1 University Plaza, Platteville, WI 53818-3099, USA

**Keywords:** hydrothermal synthesis, silver phosphate, degradation, low power white-light LED irradiation

## Abstract

This study demonstrated facile synthesis of silver phosphate (Ag_3_PO_4_) photocatalysts for the degradation of organic contaminants. Ag_3_PO_4_ microparticles from different concentrations of precursor, AgNO_3_, were produced and characterized by scanning electron microscopy, powder X-ray diffraction, and UV–visible diffuse reflectance spectroscopy. Degradation rates of methylene blue (MB) and phenol were measured in the presence of microparticles under low-power white-light light-emitting-diode (LED) irradiation and the reaction rate followed pseudo-first-order kinetics. The prepared Ag_3_PO_4_ microparticles displayed considerably high photocatalytic activity (>99.8% degradation within 10 min). This can be attributed to the microparticles’ large surface area, the low recombination rate of electron–hole pairs and the higher charge separation efficiency. The practicality of the Ag_3_PO_4_ microparticles was validated by the degradation of MB, methyl red, acid blue 1 and rhodamine B under sunlight in environmental water samples, demonstrating the benefit of the high photocatalytic activity from Ag_3_PO_4_ microparticles.

## 1. Introduction

Advanced oxidation processes (AOPs) have gained considerable attention for the treatment of organic dyestuffs polluted environmental water matrices [1,2,3,4]. Among the various AOPs, heterogeneous photocatalysis, which employs semiconductor-based photocatalysts, has been the subject of numerous studies on due to its ability to decompose organic compounds under solar light irradiation [5,6,7,8,9]. For example, the heterogeneous photocatalyst TiO_2_ is considered an excellent material for water treatment because it is cheap, nontoxic and abundant in nature [5,6,10,11]. However, its inefficient photocatalytic degradation and antibacterial activity render it non-ideal for practical applications because of the rapid recombination of its photogenerated electron–hole pairs leading to high operating costs [12]. Furthermore, as a material with wide band gap, the absorption peak of TiO_2_ is in the ultraviolet (UV) region (3.2 eV), inferring that TiO_2_ utilizes only a small proportion of solar energy. To overcome the limitation of TiO_2_, two synthetic strategies have been proposed. One is to modify the narrow the wide band gap of TiO_2_ by doping it with other materials, but this method often requires complicated synthetic procedure [13,14,15]. The other is to synthesize novel semiconductor-based photocatalysts, other than TiO_2_, capable of absorbing visible light with relative simple synthetic route.

In 2010, Yi et al. synthesized Ag_3_PO_4_ with high photo-oxidative activity for O_2_ evolution from water and the decomposition of organic dyes under visible-light irradiation [16]. The photodegradation rate of organic dyes using this novel photocatalyst is 10 folds higher than that using other visible-light-responsive photocatalysts such as BiVO_4_ and N-doped TiO_2_. Since 2010, studies have focused on the synthesis of different-morphology Ag_3_PO_4_ photocatalysts and the evaluation of their photocatalytic activity [17,18,19,20,21,22,23,24,25,26]. For example, Hsieh et al. proposed shape-controllable synthesis of Ag_3_PO_4_ crystals and examined their facet-dependent optical properties, photocatalytic activity and electrical conductivity [27]. Ag_3_PO_4_ cubes have shown more photocatalytically active than rhombic dodecahedra at degrading methyl orange whereas tetrahedral Ag_3_PO_4_ is inactive. Furthermore, the effects that synthesis temperature has on the morphology and photocatalytic performance of Ag_3_PO_4_ were demonstrated by Song [28]. They concluded that increasing the synthesis temperature to 120 °C increased the ratio of exposed (110) facets and produced crystals with higher photocatalytic efficiency over rhodamine B under visible-light irradiation. Another study focused on the effects of annealing temperature and time on the structure, morphology and photocatalytic activity of Ag_3_PO_4_ microparticles [29]. The results revealed that neither the crystalline phase nor photocatalytic activity decreased with annealing temperature, even when the Ag_3_PO_4_ powders were annealed at 400 °C for 30 min. The excellent photocatalytic activity of the sintered samples in that study were speculated to arise from high crystal quality. Although Ag_3_PO_4_ is a promising candidate for use in environmental remediation, the large amount of Ag required to produce it and its low structural stability strongly limit its practical applications [30,31,32].

In the present study, we examined the photocatalytic activity of Ag_3_PO_4_ with different amounts of AgNO_3_ through a sonochemical (20 kHz, 600 W for 30 min) and hydrothermal method (180 °C for 24 h). The photocatalytic degradation activity of the as-prepared Ag_3_PO_4_ microparticles was evaluated according to their degradation of methylene blue (MB) and phenol under low-power white-light light-emitting-diode (LED) irradiation (5 W). Finally, we propose the possible photodegradation mechanism by using the Ag_3_PO_4_ microparticles.

## 2. Materials and Methods

### 2.1. Chemicals

All chemicals were of analytical grade and the highest purity available. AgNO_3_, Na_3_PO_4_, HNO_3_, phenol, p-benzoquinone, tert-butanol (t-BuOH), and ethylenediamine tetraacetate (EDTA) were purchased from Sigma-Aldrich (St. Louis, MO, USA). MB, methyl red (MR), acid blue 1, and rhodamine B (RhB) were purchased from Invitrogen (Eugene, OR, USA). Milli-Q deionized water was used to prepare all solutions in all experiments.

### 2.2. Preparation

Ag_3_PO_4_ microparticles (S1–S4) were prepared through a sonochemical and hydrothermal method. In a typical synthesis procedure, AgNO_3_ (S1: 1.25, S2: 7.5, S3: 12.5, and S4: 25.0 mmol) was dissolved in 45 mL of deionized water. The prepared Na_3_PO_4_ solution (2.50 mmol, 5 mL) was then added to the preceding solution under ultrasonic irradiation at room temperature. After 30 min of ultrasonic irradiation (Misonix, Inc., Farmingdale, NY, USA: XL-2020, 20 kHz, 600 W), the mixture was transferred into a Teflon-lined stainless steel autoclave. The autoclave was sealed and heated in an electric oven at 180 °C for 24 h. After the autoclave naturally cooled to room temperature, the precipitates were centrifuged and washed three times with ethanol and deionized water, and then dried in vacuum at 50 °C for 8 h.

### 2.3. Characterization

The crystalline phases of the Ag_3_PO_4_ microparticles were determined through X-ray diffraction (XRD) using a SMART APEX II X-ray diffractometer (Bruker AXS, Billerica, MA, USA) with Cu Kα radiation (*λ* = 0.15418 nm). The morphologies of the Ag_3_PO_4_ microparticles were observed through a HITACHI S-4800 scanning electron microscope (SEM) (Hitachi high technologies corporation, Tokyo, Japan) operating at 15 kV. UV–visible diffuse reflectance spectra (UV-Vis-DRS) of the Ag_3_PO_4_ microparticles were collected from an Evolution 2000 UV–Vis spectrometer (Thermo Fisher Scientific Inc., Madison, WI, USA) with BaSO_4_ employed as the reference. The Brunauer–Emmett–Teller (BET) specific surface area of the Ag_3_PO_4_ microparticles was characterized using a PMI C-BET 201A system (Porous Materials Inc., Ithaca, NY, USA).

### 2.4. Photocatalytic Degradation Activity

The Ag_3_PO_4_ microparticles (S1–S4) were used to degrade MB and phenol under white-light LED irradiation (5 W, PCX-50C, Beijing perfectlight technology co. LTD, Beijing, China), and their photocatalytic activity was evaluated based on our earlier reports with modifications [33,34,35,36,37]. Prior to irradiation, 50 mL of 5 mg/L MB aqueous solution or 10 mg/L phenol solution containing 50 mg of photocatalyst was stirred in the dark for 30 min. During irradiation from the bottom with constant magnetic stirring, 3 mL of suspension was taken and centrifuged to remove the photocatalyst. The MB or phenol content in the filtrate was determined colorimetrically at 665 nm and 270 nm, respectively, by using a using a Synergy H1 Hybrid Multi-Mode Microplate Reader (Biotek Instruments, Inc., Winooski, VT, USA). Furthermore, the amount of total organic carbon was measured during degradation using an elementar Acquray TOC analyzer (Elementar Analysensysteme GmbH, Langenselbold, Germany). Similar procedures were performed for various dyestuffs (MR, RhB and acid blue 1) and environmental water samples (lake water and pond water), except for the use of solar irradiation.

### 2.5. Study of Recombination Rate of Electron–Hole Pairs and Charge Separation Efficiency

The recombination rate of the electron–hole pairs and charge separation efficiency for the Ag_3_PO_4_ microparticles (S1–S4) were evaluated based on our earlier reports [33,34,35,36,37]. For determination of recombination rate of electron–hole pairs, photoluminescence spectra were produced using a Varian Cary Eclipse fluorescence spectrometer (Agilent Technologies, Inc., Santa Clara, CA, USA). A slurry containing a photocatalyst (5 mg) and methanol (5 mL) was coated on the indium tin oxide (ITO) glass. The coated ITO glass was then heated on the hotplate at 50 °C for 30 min to completely remove the methanol. This coated ITO glass was used as an anode electrode, a platinum foil was used as a cathode electrode, and 0.1 M aqueous Na_2_SO_4_ solution was used as a supporting electrolyte. Photocurrents, which used to evaluate the charge separation efficiency, were measured using a CHI-6122E electrochemical analyzer (CH instruments, Inc., Austin, TX, USA) at room temperature.

### 2.6. Scavenger Test

The scavenger test was used to determine the major reactive species involved in the MB degradation. The procedure was similar to that the photocatalytic activity procedure [33,34,35,36,37]. Prior to adding a photocatalyst, hole-, oxygen radical- and hydroxyl radical- scavengers (each 10.0 µM) were added to the MB solution.

## 3. Results and Discussion

### 3.1. Crystalline Phase and Morphology

**Figure 1:**
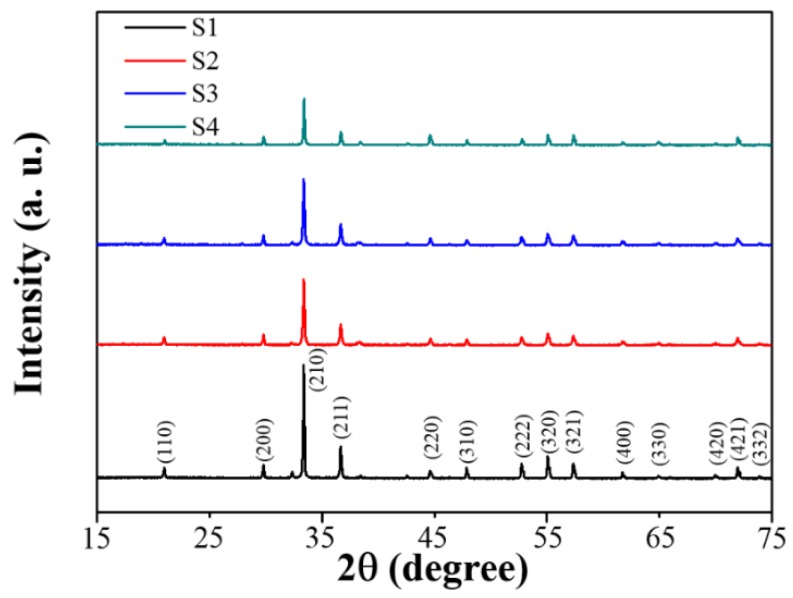
XRD patterns of the materials synthesized from different AgNO_3_ concentrations (S1: 1.25 mmol, S2: 7.5 mmol, S3: 12.5 mmol, and S4: 25.0 mmol of AgNO_3_.) under sonochemical process (600 W) at 25 kHz for 30 min with the hydrothermal process at 180 °C for 24 h.

Different concentration effect on silver nitrate was examined by XRD and SEM to reveal their influence on crystalline phase and morphology of the Ag_3_PO_4_ microparticles. The XRD patterns of the various Ag_3_PO_4_ microparticles prepared from varying AgNO_3_ concentration are presented in Figure 1.

The main diffraction peaks for all the types of Ag_3_PO_4_ microparticle can be indexed to a body-centered cubic structure (Joint Committee on Powder Diffraction Standards 06-0505), indicating that the AgNO_3_ concentration have no influence on the crystalline phase of the Ag_3_PO_4_ microparticles. 

**Figure 2:**
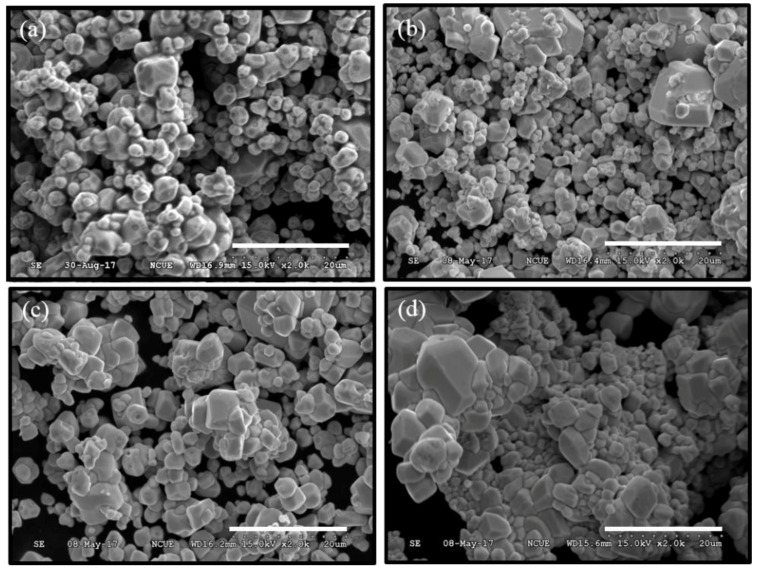
SEM images of the Ag_3_PO_4_ microparticles synthesized from different AgNO_3_ concentrations under sonochemical (600 W) at 25 kHz for 30 min and hydrothermal process at 180 °C for 24 h: (**a**) 1.25 mmol (S1), (**b**) 7.5 mmol (S2), (**c**) 12.5 mmol (S3), and (**d**) 25.0 mmol (S4) of AgNO_3_. Scale bar: 20 µm.

Figure 2 shows SEM images of the Ag_3_PO_4_ microparticles, which reveal clear differences in the morphology and size of particles. The Ag_3_PO_4_ microparticle (S1), which was prepared by mixing 1.25 mmol of AgNO_3_ with 2.5 mmol of Na_3_PO_4_ in 40.0 mL DI-water through the sonochemical and hydrothermal process, was constructed from spherical particles with a diameter of 4.5 ± 1.7 µm (Figure 1a). As the AgNO_3_ content was increased gradually (7.50, 12.5 and then 25.0 mmol), the Ag_3_PO_4_ microparticles became irregular in shape and increased in size (Figure 1b: 7.3 ± 4.3 µm; Figure 1c: 11.3 ± 2.9 µm; and Figure 1d: 13.6 ± 5.0 µm). Because AgNO_3_ was the precursor, a high concentration of AgNO_3_ enhanced the growth rate of the Ag_3_PO_4_ particles; thus, the morphology changed from spherical to irregular, dependent on the AgNO_3_ content.

### 3.2. Photophysical Properties and Specific Surface Area

**Figure 3:**
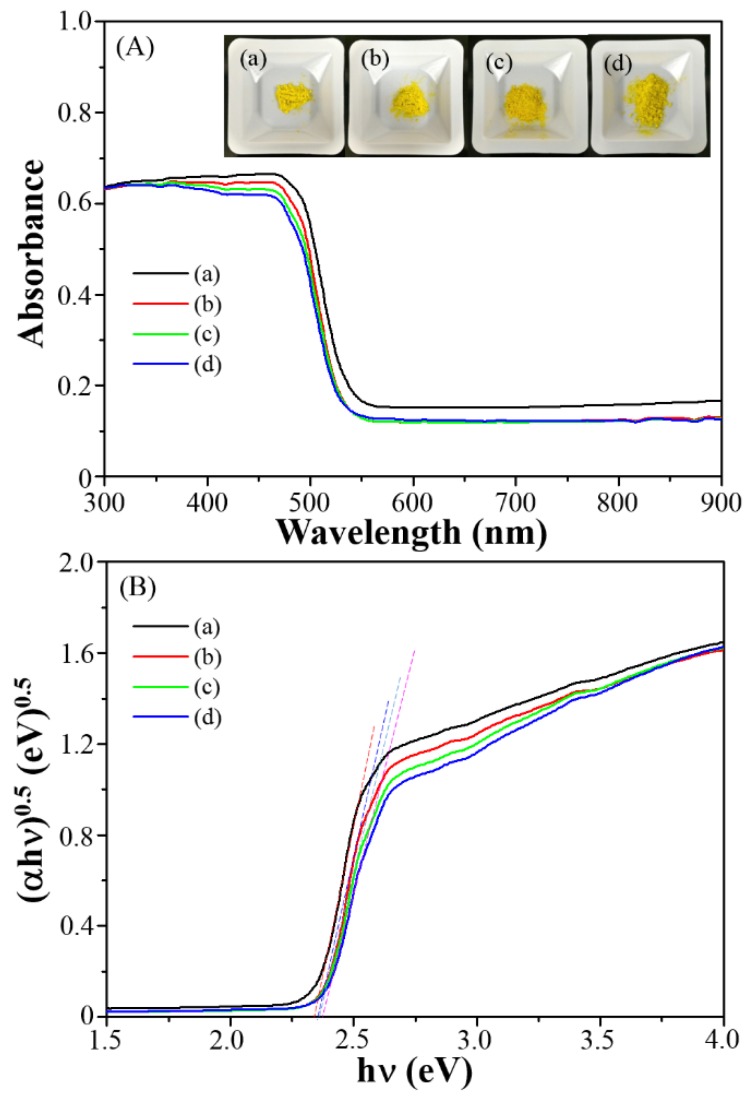
(**A**) UV–visible diffuse reflectance spectra (UV-Vis-DRS) and (**B**) (αhν)^0.5^ versus hν curves of Ag_3_PO_4_ (a) S1, (b) S2, (c) S3, and (d) S4. Inset: photographs of the corresponding materials.

Figure 3A displays the UV-Vis-DRS spectra of the Ag_3_PO_4_ microparticles, which clearly reveal that the yellow Ag_3_PO_4_ microparticles absorbed from UV to visible light range. The square root of the absorption coefficient was linearly correlated with energy, signifying that the absorption edges of all the Ag_3_PO_4_ microparticles were due to indirect transitions (Figure 3B). The energy bandgaps (Eg) of the microparticles were determined according to Tauc Plots [38]. Given that n for Ag_3_PO_4_ is 4 because of indirect transitions, the corresponding *E_g_* of the Ag_3_PO_4_ microparticles were found from the x-axis intercept of the curve tangent in the plot of (αhν)^0.5^ versus hν (Figure 3B) [37]. The estimated band gaps of the Ag_3_PO_4_ microparticles were approximately in the range 2.32–2.41 eV (Table 1) [27]. The difference in band gap of microspheres generated from various AgNO_3_ concentration may have caused different charge separation efficiency and recombination rate of the photogenerated electron–hole pairs, influencing photocatalytic activities of microspheres. The conduction band (CB) and valence band (VB) positions in the Ag_3_PO_4_ microparticles were determined according to the following equation: *E_VB_* = 1.46 + 0.5*E_g_,* where *E*g is the band gap [27,38]. Through this equation, *E_VB_* and *E_CB_* for the Ag_3_PO_4_ microparticles were calculated (Table 1).

The BET specific surface area of the Ag_3_PO_4_ microparticles was determined using nitrogen adsorption–desorption measurements; the corresponding values are listed in Table 1. Apparently, BET-determined surface area decreased with an increase in AgNO_3_ concentration. This result is consistent with the size of the Ag_3_PO_4_ microparticles from SEM images. It is noteworthy to learn the difference in surface area may affect the extent of interaction between the photocatalyst and an organic pollutant, which may also cause different photocatalytic level.

### 3.3. Photocatalytic Degradation, Mechanism and Applications

**Figure 4:**
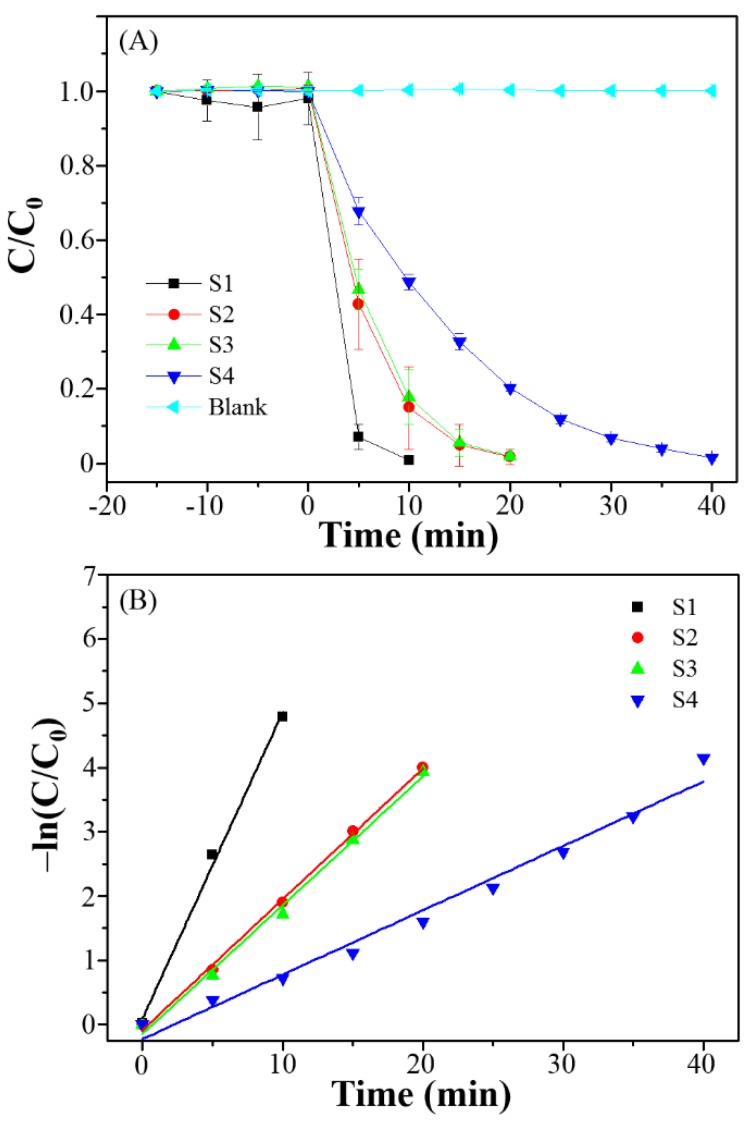
(**A**) Reduction of the concentration of methylene blue (MB) (C/C_0_) with time and (**B**) corresponding rate constant of MB degradation by Ag_3_PO_4_ (S1–S4) under white-light LED irradiation (5 W).

The photocatalytic efficiency of the Ag_3_PO_4_ microparticles during MB degradation under white-light LED irradiation (5 W) was evaluated. Figure 4A plots the MB concentration ratio (C/C_0_, where C_0_ represents initial concentration and C represents concentration at time t) with irradiation time when different photocatalyst sample, S1–S4, was used. To make sure the Ag_3_PO_4_ microparticles were photocatalytically active, an experiment of direct MB photolysis in the absence of photocatalyst was performed under the same conditions. The MB concentration barely changed in the control experiment as the irradiation time increased (cyan curve). Under white-light LED irradiation for 10 min, S1 was clearly the most photocatalytically active (>99% degradation) among all other samples, S2–S4. Interestingly, Ag_3_PO_4_ microparticles prepared without hydrothermal process also exhibited >99% degradation within 10 min. However, they were not stable during the photocatalytic process (discussion later) because of photocorrosion [31,32,39]. In this study, S1 was the optimal candidate for MB degradation. According to the data in Figure 4A, the MB degradation was fitted as a pseudo-first-order reaction (Figure 4B). The rate constant and the half-life for MB degradation by the Ag_3_PO_4_ microparticles were determined (the corresponding values are listed in Table 2). Notably, S1 resulted in the highest rate constant and the shortest half-life for MB degradation. This may be because S1 possessed a large specific surface area, which resulted in high photodegradation activity.

**Figure 5:**
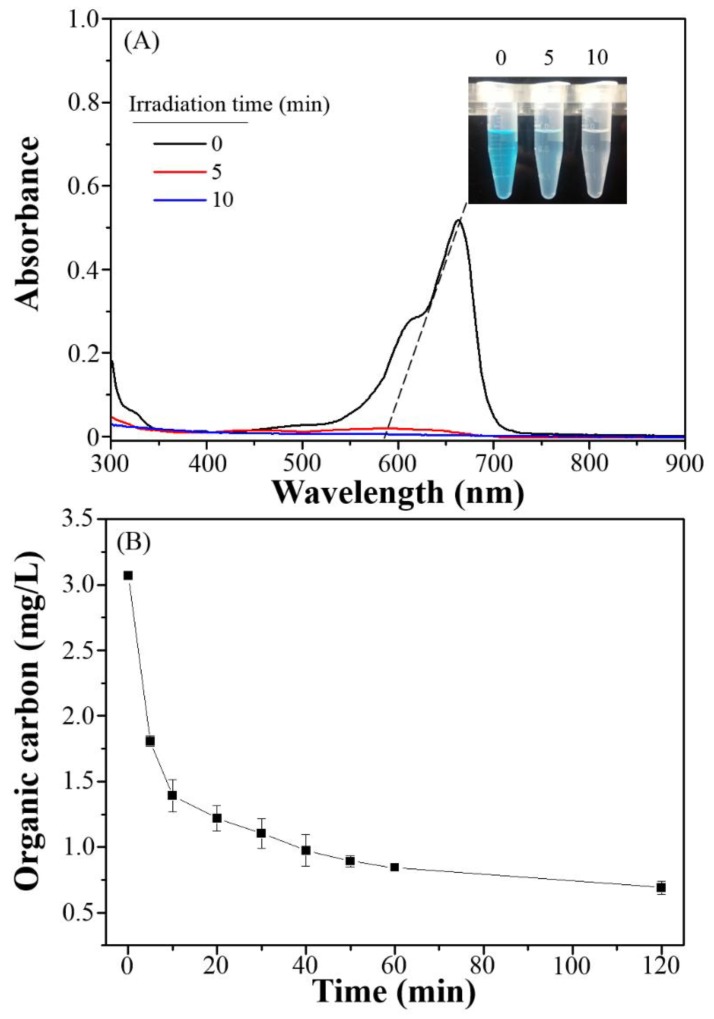
(**A**) Temporal evolution of the spectrum and (**B**) decomposition of MB by Ag_3_PO_4_ (S1) under white-light light-emitting-diode (LED) irradiation (5 W).

Figure 5A shows the temporal evolution of the spectrum with MB degradation by S1. According to the literature, chromophore cleavage is analogous to the competitive photodegradation reaction involved in the organic pollutants decomposition [37]. In this study, the MB absorption at 665 nm decreased with an increase in the irradiation time. Moreover, a slight hypochromic shift was found in the characteristic absorption of MB in the presence of S1. Thus, the cleavage of the MB chromophore appears to predominate in Ag_3_PO_4_-photocatalytic decomposition systems, supported by the results from Figure 5B, which plots the temporal evolution of the total organic carbon content with MB degradation over S1. Although the colour of the MB solution disappeared under white-light LED irradiation for 10 min, the total organic carbon content only decreased to 1.39 mg/L (approximately decomposition 54.7% of original concentration). The photocatalytic decomposition of MB by S1 was increased as high as 71.0% (0.89 mg/L) under white-light LED irradiation for 120 min.

**Figure 6:**
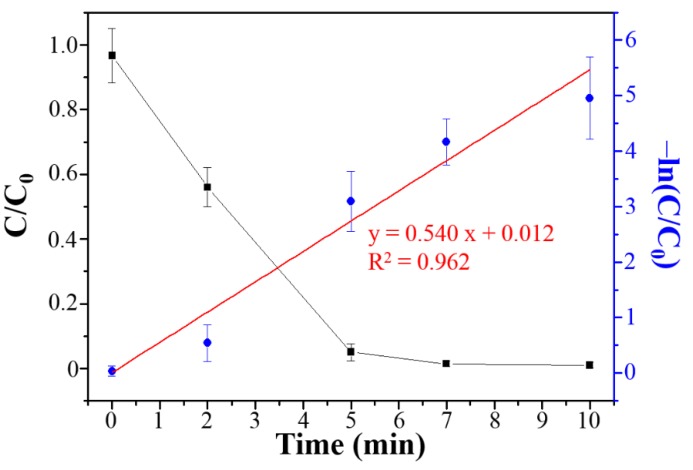
Reduction of the concentration of phenol (C/C_0_) with time and the corresponding rate constant of phenol degradation by Ag_3_PO_4_ (S1) under white-light LED irradiation (5 W).

In order to exclude the MB sensitization under white-light LED irradiation, the photocatalytic activity of S1 was also evaluated by degradation of colorless phenol. The change in phenol concentration with irradiation time when S1 was used as shown in Figure 6. It is found that the concentration of phenol decreases with the irradiation time. The rate constant for degradation of phenol by S1 was 0.540 min^−1^.

**Figure 7:**
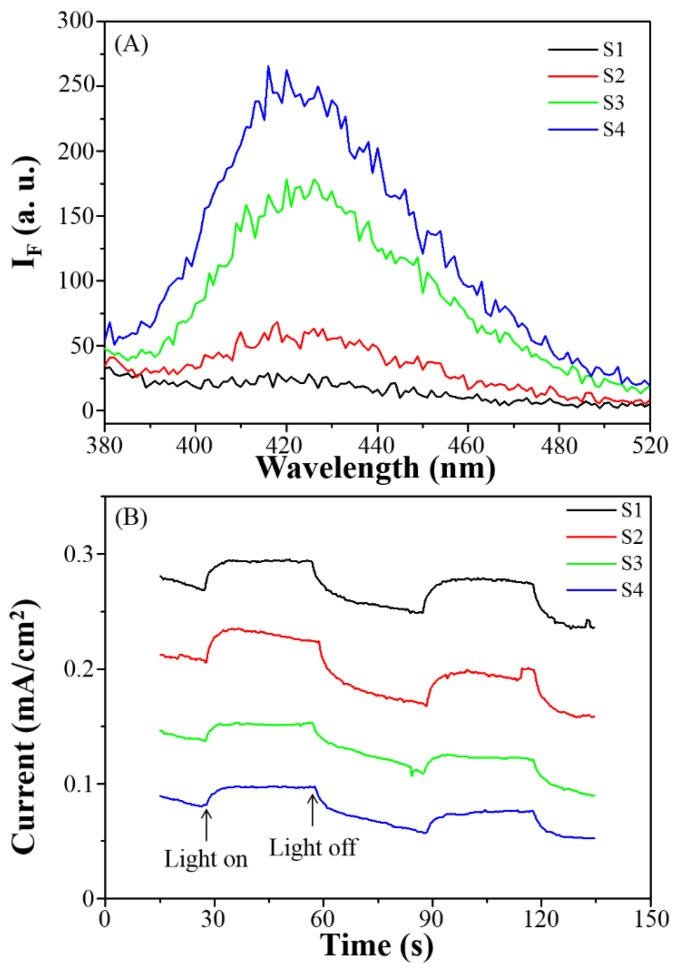
(**A**) Photoluminescence at λ_ex_ = 365 nm and (**B**) photocurrents spectra measured of Ag_3_PO_4_ (S1–S4).

Besides the specific surface area of microparticles, the recombination rate of photogenerated electron–hole pairs and charge separation efficiency can affect the photocatalytic activity. Recombination of electron–hole pairs can release energy in the form of luminescence; thus, a low recombination rate suggests a lower luminescence intensity in the photoluminescence measurement and can correlate to higher photocatalytic activity of microspheres. Therefore, the luminescence spectra of the Ag_3_PO_4_ microparticles were measured at an excitation wavelength of 365 nm (Figure 7A). The luminescence intensity of S1 was the lowest among all samples (S1–S4), implying that the electron–hole pairs in S1 recombined the slowest. This may be because the lowest AgNO_3_ concentration made Ag atoms on the Ag_3_PO_4_ microparticles surface coordinatively unsaturated (three-coordinated sites with one dangling bond) [40], inhibiting the recombination of photogenerated electrons and holes, and thus enhanced photocatalytic activity. The charge separation efficiency was evaluated through photocurrent measurements. Generally, a larger photocurrent implies higher separation efficiency and higher photocatalytic activity. The photocurrent of S1 was higher than those of other samples (Figure 7B), indicating enhanced separation of photogenerated electrons and holes. Therefore, the high photocatalytic activity of S1 was due to its large specific surface area, the low recombination rate of the photogenerated electron–hole pairs and the higher charge separation efficiency.

**Figure 8:**
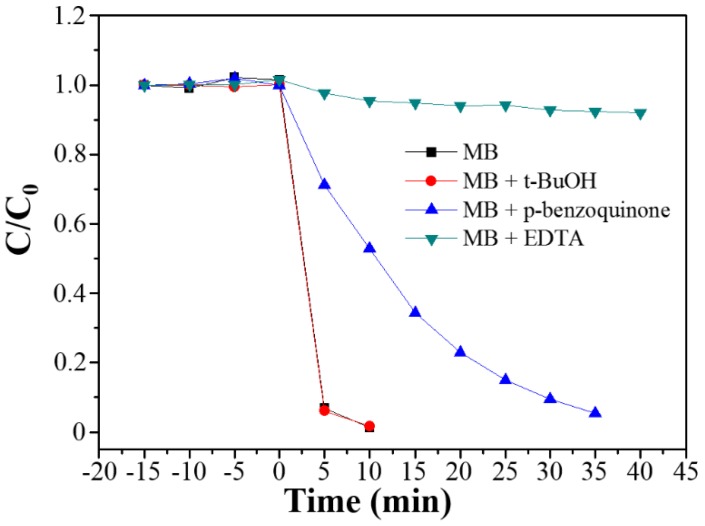
Plots of photogenerated carrier trapping for MB decolorization catalyzed by Ag_3_PO_4_ (S1).

To resolve the mechanism responsible for the degradation of MB, radical- and hole-scavenger tests were implemented to identify the main active species in the photocatalytic degradation process when S1 was used. Under white-light LED irradiation, MB degradation was deceased upon the addition of a hole scavenger (EDTA) and an oxygen radical scavenger (p-benzoquinone), as illustrated in Figure 8. This indicates that hole and oxygen radicals are the major reactive species involved in the MB decomposition.

**Scheme 1:**
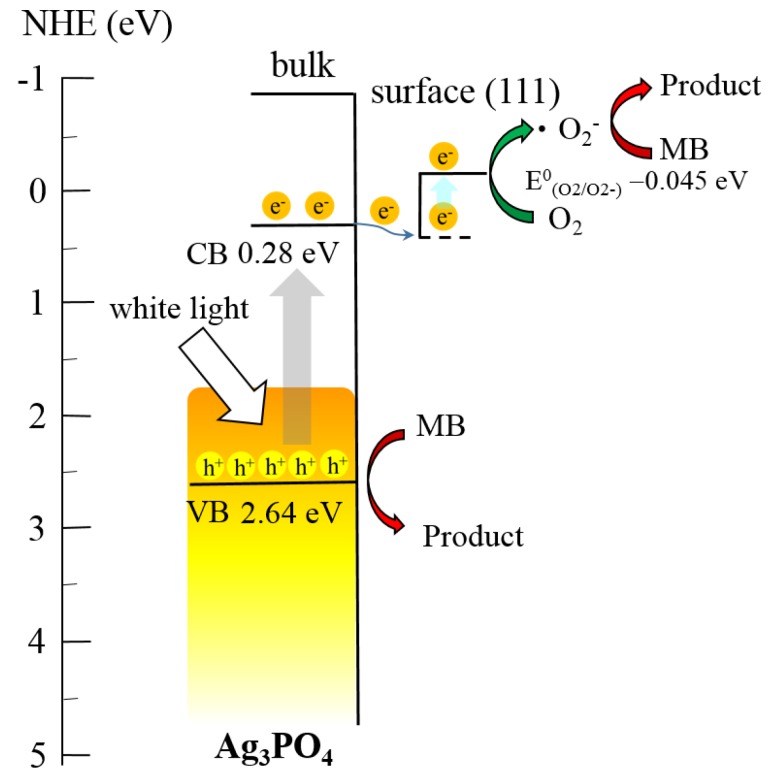
Illustration of the photocatalytic degradation mechanism of the Ag_3_PO_4_ microparticles under white-light LED irradiation.

A possible degradation mechanism is proposed in Scheme 1. When Ag_3_PO_4_ was illuminated under white-light LED irradiation, electrons in the VB were promoted to the CB, with an equal number of holes produced in the VB (Equation (1)). Then, the electrons in the CB were transferred to the (111) surface of the Ag_3_PO_4_ microparticles, which decreased the electron–hole recombination rate. The electrons on the (111) surface were unstable and could react with oxygen to produce oxygen radicals (Equations (2) and (3)) [17,20,22,23,24,25]. Therefore, holes in the VB and oxygen radicals were the major reactive species in the degradation of MB (Equations (4) and (5)).

Ag_3_PO_4_ + hν → Ag_3_PO_4_ (h^+^ + e^−^) (1)

Ag_3_PO_4_ (h^+^ + e^−^) → Ag_3_PO_4_ (h^+^_VB_) + Ag_3_PO_4_ (e^−^_CB_) (2)

Ag_3_PO_4_ (e^−^_CB_) + O_2_ → ^•^O_2_^−^ (3)

^•^O_2_^−^ + MB → oxidized products (4)

Ag_3_PO_4_ (h^+^_VB_) + MB → oxidized products (5)

**Figure 9:**
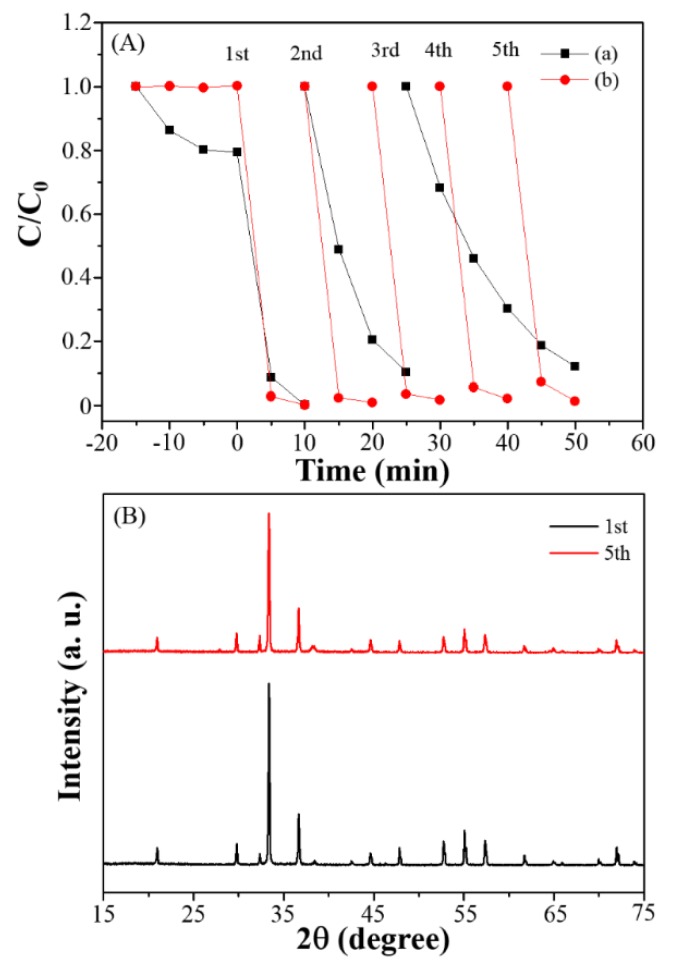
(**A**) Cycling runs of MB photocatalytic decolorization in the presence of the Ag_3_PO_4_ microparticles prepared (a) without and (b) with hydrothermal process. (**B**) XRD patterns for S1 before and after being used for five reaction cycles.

In addition to the photocatalytic efficiency of the photocatalyst, the stability of Ag_3_PO_4_ (S1) is vital for practical applications. To assess the stability and efficiency of the Ag_3_PO_4_ microparticles, a prolonged run of MB degradation was performed. After three cycles for 10 min in each cycle, a decrease in the photodegradation rate was observed when the Ag_3_PO_4_ microparticles prepared without hydrothermal process was employed (black curve in Figure 9A) while the MB photodegradation activity of S1 remained constant even after five cycles (red curve in Figure 9A), maintaining 98.7% of activity within 10 min after five cycles. This indicated that S1 had an excellent stability, which was proved by the lack of change in the XRD pattern after five photolysis cycles (Figure 9B). This may be because the hydrothermal procedure enhanced the degree of crystallinity in the microparticles [29].

**Figure 10:**
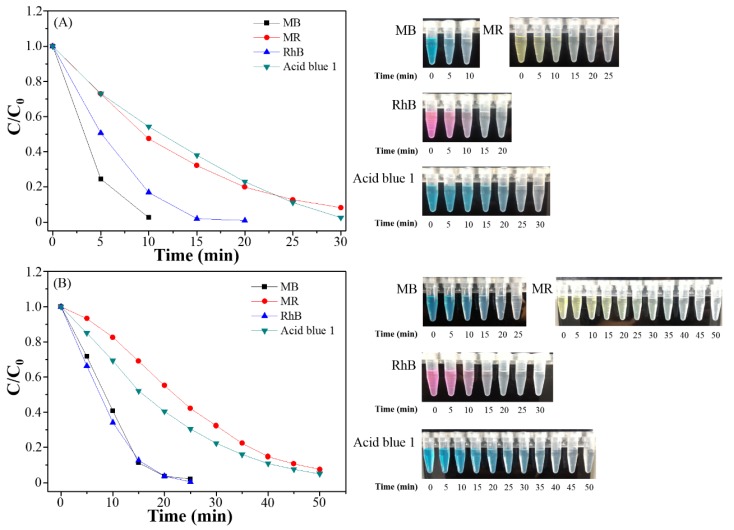
Decoloration kinetic curves and photographs of different organic dyes (MB, MR, RhB and acid blue 1, each 10 ppm), degraded using S1 in (**A**) lake water and (**B**) pond water under solar light irradiation.

To assess the practical applications of S1, various dyestuffs (MB, MR, RhB and acid blue 1) in environmental water samples (lake water and pond water) were degraded under sunlight (Figure 10). S1 exhibited excellent photocatalytic activity under sunlight for the degradation of all dyestuffs, with nearly 90% degradation within 50 min. A remarkable difference in the degradation time for all dyestuffs was observed for the environmental water samples (90% degradation within 50 min) compared with the deionized water sample (100% degradation within 10 min). This may be because the presence of anions or radical scavengers in the environmental water samples reduced the photocatalytic activity of S1. Further research on the enhancing photocatalytic activity of Ag_3_PO_4_ composites, such as those wrapped by reduced graphene oxide and g-C_3_N_4_, is underway in our laboratory.

## 4. Conclusions

The Ag_3_PO_4_ microparticles were synthesized using a combination of sonochemical and hydrothermal processes from various concentrations of AgNO_3_. The photocatalytic activity of the Ag_3_PO_4_ microparticles was evaluated through the degradation of MB under low-power white-light LED irradiation. The results reveal that S1 synthesized from 1.25 mmol AgNO_3_ in 40.0 mL DI-water displayed remarkably higher photocatalytic degradation activity (>99.8%) within 10 min than other microparticle types (S2–S4). This can be attributed to its large specific surface area, the low recombination rate of its photogenerated electron–hole pairs and the higher charge separation efficiency. This study shows that the strong photocatalytic activity of the Ag_3_PO_4_ microparticles (S1) was considered to be possible for its applications toward photodegradation of organic pollutes in environmental water samples.

## Figures and Tables

**Table 1:** Measured band gap (Eg), conduction-band (E_CB_) edge, valence-band (E_VB_) edge, and specific surface area (S_BET_) for the Ag_3_PO_4_ catalysts.

Series	Eg (eV)	E_CB_ (eV)	E_VB_ (eV)	S_BET_ (m^2^·g^−1^)
S1	2.36	0.28	2.64	0.65
S2	2.38	0.27	2.65	0.55
S3	2.39	0.27	2.66	0.48
S4	2.41	0.26	2.67	0.36

**Table 2:** Pseudo-first-order rate constants and half-life for photocatalytic decoloration of MB by various Ag_3_PO_4_ photocatalysts.

Series	First-Order Kinetic Equation	k (min^−1^)	R^2^	Half-Life (min)
S1	y = 0.478x + 0.100	0.478	0.9932	1.45
S2	y = 0.203x − 0.087	0.203	0.9978	3.41
S3	y = 0.200x − 0.147	0.200	0.9931	3.47
S4	y = 0.100x − 0.224	0.100	0.9783	6.93
